# Impact of Microbial Composition of Cambodian Traditional Dried Starters (Dombea) on Flavor Compounds of Rice Wine: Combining Amplicon Sequencing With HP-SPME-GCMS

**DOI:** 10.3389/fmicb.2018.00894

**Published:** 2018-05-08

**Authors:** Sokny Ly, Hasika Mith, Cédric Tarayre, Bernard Taminiau, Georges Daube, Marie-Laure Fauconnier, Frank Delvigne

**Affiliations:** ^1^Terra Research Centre, Microbial Processes and Interactions, University of Liège, Gembloux Agro-Bio Tech, Gembloux, Belgium; ^2^Department of Chemical Engineering and Food Technology, Institute of Technology of Cambodia, Phnom Penh, Cambodia; ^3^Food Science Department, Faculty of Veterinary Medicine, Fundamental and Applied Research for Animal and Health, University of Liège, Liège, Belgium; ^4^General and Organic Chemistry, Université de Liège – Gembloux Agro-BioTech, Gembloux, Belgium

**Keywords:** rice wine fermentation, microbial communities, dried starter, SPME-GCMS, amplicon sequencing

## Abstract

Dombae is a traditional ferment starter which has been used for starchy based wine production in Cambodia. However, the production technology of rice wine in Cambodia is not optimized. The current study aimed to investigate the microbiota associated in five ferment starters and the effect of a traditional fermentation process using a metagenomics sequencing analysis and HS-SPME-GCMS for the characterization of the aromatic profiles at the end of fermentation. Most of bacteria identified in this study were lactic acid bacteria including *Weissella cibaria, Pediococcus sp. MMZ60A, Lactobacillus fermentum*, and *Lactobacillus plantarum*. *Saccharomyces cerevisiae* and *Saccharomycopsis fibuligera* were found to be abundant yeasts while the only amylolytic filamentous fungus was *Rhizopus oryzae*. A total of 25 aromatic compounds were detected and identified as esters, alcohols, acids, ketones and aldehydes. The alcohol group was dominant in each rice wine. Significant changes were observed at the level of microbial communities during fermentation, suggesting microbial succession for the assimilation of starch and subsequently assimilation of fermentation by-products leading to the production of flavor compounds. At this level, the presence of *Weissella, Pediococcus*, and *Lactobacillus* genus was strongly correlated with most of the flavor compounds detected.

## Introduction

The fermentation process allows to preserve and to enhance the nutritional value of food resources. All over the world, human societies without exception found the way of making fermented beverages from sugar sources available in their local habitats. Similarly, Cambodian people apply the traditional fermentation beverage process to many raw materials such as rice, cassava and other starchy resources. Rice-based fermented beverage are called rice wine in most Asian countries such as following: in India (Jeyaram et al., 2008), in Thailand (Chuenchomrat et al., 2008), in China (Wang et al., 2014), in Korea (Kim et al., 2011), in Vietnam (Dung et al., 2005). Rice-wine is a generic name referring to alcoholic beverages made from cereals, mainly rice.

Beside non-sticky rice, red rice is also used to produce wine (red rice wine), which is particularly desired for its brown-red color and special fruity aromas. Its uncommon characteristics in comparison to the colorless wine from white rice make it much more attractive. Furthermore, red rice contains polyphenols and anthocyanins, which have been reported to be highly effective cholesterol treatment in the human body and to inhibit the growth of tumor cells (Sompong et al., 2011). Microbial ferment starters, under the form of dried powders or hard ball made from starchy cereals, are used to induce alcoholic fermentation. These starters' preparations have different names such as *Loogpang* in Thailand, *Bubod* in Philippines, *Marcha* in India and Nepal (Sha et al., 2017), *Ragi* in Indonesia, Chinese yeast or *Chiuchu* in Taiwan (Ellis, 1985), *Nuruk* in Korea (Park et al., 2014) and *medombae* or *dombae* in Cambodia (Chim et al., 2015). Both starter preparation and rice wine fermentation were first made in uncontrolled conditions and with different methods, depending on the wine maker. The principle of rice wine production consists of saccharification of steamed starchy resource by fungi under solid state fermentation and by yeasts under submerged alcoholic fermentation (Blandino et al., 2003; Sujaya et al., 2004; Dung et al., 2007). These traditional processes in Cambodia lack research and optimization in the field of food technology. This optimization requires the food safety, the control of nutritional value, the improvement of production methods, the sustainable quality and the reduction of production costs. Rice wine producers regularly met the problems of a low yield of rice wine and the inconsistency of quality in terms of taste and flavor. The nature of microbial communities in Cambodian traditional starters, their interactions and their contributions to the synthesis of aromas during fermentation are still widely unknown. Several studies were previously focused on the microbial diversity in ferment starters (Ercolini, 2004; Jeyaram et al., 2008; Thanh et al., 2008; Lv et al., 2012, 2015; Chao et al., 2013; Xie et al., 2013; Luangkhlaypho et al., 2014; Wang et al., 2014; Sha et al., 2017). A very few studies were investigated on the ferment starters and the fermentation process in Cambodia. Therefore, the objective of this study was not only to investigate the composition of microbial communities in dried starters but also their evolution after the fermentation process. Furthermore, the aromatic profiles of each rice wine were analyzed to understand the different flavors of rice wines depending on the type of starter.

## Materials and methods

### Sample collection

The Cambodian traditional starters were produced through different methods. The starters were collected from five different regions in Cambodia and labeled as DBB, DCK, DOB, DOS, and DPK. The red rice used in this study was purchased from only one growing region and freshly harvested in November 2015 (rice harvesting season in Cambodia). The samples were stored in the laboratory at 4° or −20°C for further analyses.

### Fermentation of red rice

The laboratory scale processing of red rice wine production was adapted from the traditional process by local rice wine producers. Briefly, 100 g of red rice were soaked in distilled water for 3 h. A volume of 100 mL of distilled water was then added and steamed in an autoclave at 120°C for 20 min. The gelatinized rice paste was cooled to room temperature, then inoculated and mixed with 2% of traditional dried starter before being incubated at 30°C. After a solid-state aerobic fungal fermentation of 3 days, an additional volume of 100 mL of sterilized water was added to boost the alcoholic fermentation for other 7 days more in the same flask. The fermented rice mashes were homogenized and the sampling was made every 24 h.

### Sugar and ethanol analysis by HPLC

The concentrations of maltotriose, maltose, glucose and ethanol were determined using RID-HPLC (Agilent 1100 series, Agilent Technologies). A volume of 5 μL was injected, in duplicate, through a Rezex ROA-Organic Acid column (300 × 7.8 mm) with 5 mM H_2_SO_4_ as mobile phase at a flow rate of 0.6 mL/min at 60°C.

### Aromatic compounds analysis by HP-SPME-GC-MS

Rice wine mash was collected to analyze the aromatic compounds immediately after 10 days of fermentation. A 50 μm DVB/CAR/PDMS (Supleco, Bellofonte, PA, USA) was used as the extract fiber coating to perform the Headspace Solid-Phase Micro-extraction. The fiber was conditioned according to the manufacturer's instructions. A volume of 5 mL of rice wine sample with 30% NaCl and 1 μL Octan-2-ol (80.2 mg/L prepared in absolute ethanol) as internal standard were added into a 20 mL screw cap glass vial containing a magnetic stirring bar. The final concentration of octan-2-ol was 1.6 mg/L. The fiber was exposed to the sample containing vial for 30 min at 60°C, after 30 min of equilibration. For all experiments, the desorption was done in the splitless mode using helium at a flow rate of 50 mL/min. The identification of the extracted analytes was performed in an Agilent 6890 GC with a VF-WAXms capillary column (30 mm, 0.25 mm I.D., 0.25 mm film thickness, Agilent Technologies). The carrier gas was helium at a flow rate of 1.9 mL/min. The injector temperature was at 250°C. The mass detector operated in the electron impact mode at 70 eV in a range from 35 to 400 amu, and the ion source temperature was set at 230°C. The oven temperature was held at 35°C for 2 min, raised at 5°C/min to 155°C, then raised to 250°C at a rate of 20°C/ min, and held at 250°C for 10 min. The aromatic components were identified by comparison of their Retention Indices with data reported in the literature and their mass spectra to the NIST 05 data base (matching quality higher than 90%). The Retention Indices (RI) of unknown compound were calculated by the retention time of a series of alkanes (C5-C35). A semi-quantification of the volatile compounds was performed using octan-2-ol as the internal standard. The quantification of each compound was performed if the peak represented more than 1% of the total area. The results were reported in the mean value of three biological replication of rice wine mash.

### 16S and 28S rDNA pyrosequencing

Total DNA was extracted from ferment starter and rice wine mush with the DNEasy Blood and Tissue kit (QIAGEN Benelux BV, Antwerp, Belgium) following the manufacturer's recommendations. For each sample, the pyrosequencing was conducted in two biological replications. The DNA was eluted into DNase/RNase-free water and its concentration and purity were evaluated by absorbance measurement using the NanoDrop ND-1000 spectrophotometer (NanoDrop ND-1000, Isogen). PCR-amplification of the V1-V3 region of the 16S rDNA was performed. Primers targeting the 16S rRNA gene fragments E9-29, 5′-GAGAGTTTGATCATGGCTCAG-3′, and E514-530, 5′-ACCGCGGCTGCTGGCAC-3′ (Baker et al., 2003) were used for their theoretical ability to generate lowest possible amplification capability bias among the various bacteria. The oligonucleotide design included 454 Life Sciences' A or B sequencing titanium adapters (Roche Diagnostics) and multiplex identifiers (MIDs) fused to the 5′ end of each primer. PCR was performed in the following condition: the amplification mix contained 5 U FastStartHigh Fidelity DNA polymerase (Roche Diagnostics, Vilvoorde, Belgium), 1 × enzyme reaction buffer, 200 μM dNTPs (Eurogentec, Liège, Belgium), each primer at 0.2 μM, and 100 ng genomic DNA in a final volume of 100 μL. Thermocycling conditions were denaturation at 94°C for 15 min followed by 25 cycles of 94°C for 40 s, 56°C for 40 s, 72°C for 1 min, and a final 7 min elongation step at 72°C. The amplification was carried out on a Mastercycler ep Gradient thermocycler (Eppendorf, Ham- burg, Germany). The PCR products were electrophoresed through a 1% agarose gel and the DNA fragments were plugged out and purified with the SV PCR Purification Kit (Promega Benelux). The quality and quantity of the products was assessed with a Picogreen dsDNA quantification assay. All amplicons was sequenced with the Roche GS-Junior Genome Sequencer (Roche, Vilvoorde, Belgium). Positive control using DNA from 20 defined bacterial species and a negative control (from the PCR step) were included in the sequencing run. The same procedure was applied for fungi, except that a 500-pb fragment of the 28S rRNA gene was amplified and sequenced with the following primers: NL-1, 5′-GCATATCAATAAGCGGAGGAAAAG-3′, and NL-4, 5′-GGTCCGTGTTTCAAGACGG-3′ (Kurtzman and Robnett, 1997). All libraries were run in the same titanium pyrosequencing reaction using Roche multiplex identifiers, and amplicons were sequenced using the Roche GS-Junior Genome Sequencer instrument (Roche).

### Bioinformatics analysis of the pyrosequencing products

The 16S and 28S rDNA sequence reads were processed using the MOTHUR software package (Schloss et al., 2009). The quality of all the sequence reads was assessed by using the PyroNoise algorithm implemented in MOTHUR and the data were screened according to the following criteria: minimal length of 425 bp, an exact match to the barcode, and one mismatch allowed for the proximal primer. ChimeraSlayer was used to check the sequences for the presence of chimeric amplification (Haas et al., 2011). The resulting reads were compared with a reference dataset (derived from the SILVA database) of full-length rRNA sequences implemented in MOTHUR. The final reads were clustered into operational taxonomic units (OTU) with the nearest neighbor algorithm using MOTHUR with a 0.03 distance unit cutoff. When taxonomic identification was below the 80% threshold, the taxonomic level was labeled with the first defined level from higher level followed by the term “_unclassified.” Population structure and community membership were assessed with MOTHUR using distance matrices based on the Jaccard index (a measure of community membership; which considers the number of shared OTUs but not their abundance) and the Yue and Clayton measure of dissimilarity (a measure of community structure which considers shared OTUs and their relative abundances) (Eshar and Weese, 2014). Richness estimation (Chao1 estimator) (Chao and Bunge, 2002), microbial biodiversity [non-parametric (NP) Shannon diversity index] (Chao and Shen, 2003), and the population evenness (Shannon evenness) (Mudler et al., 2004) were calculated using MOTHUR. Chao 1 estimator was used to estimate the richness of the detected species (OTUs) in a sample (Delcenserie et al., 2014).

### Statistical analysis

Five percentage from each strain presented in dried starter and corresponding flavor compounds were analyzed their correlation with the significant level 95% by using SPSS v.23. Only the 24 bacterial strains and two yeast species were analyzed due to limited value of others strains and those strains were not observed after fermentation. While the correlation with significant *p*-value were observed, those values were imported to Cytoscape Network software to visualize their interrelationship.

## Results

### Bacterial communities in cambodian traditional dried starters

**Figure 1:**
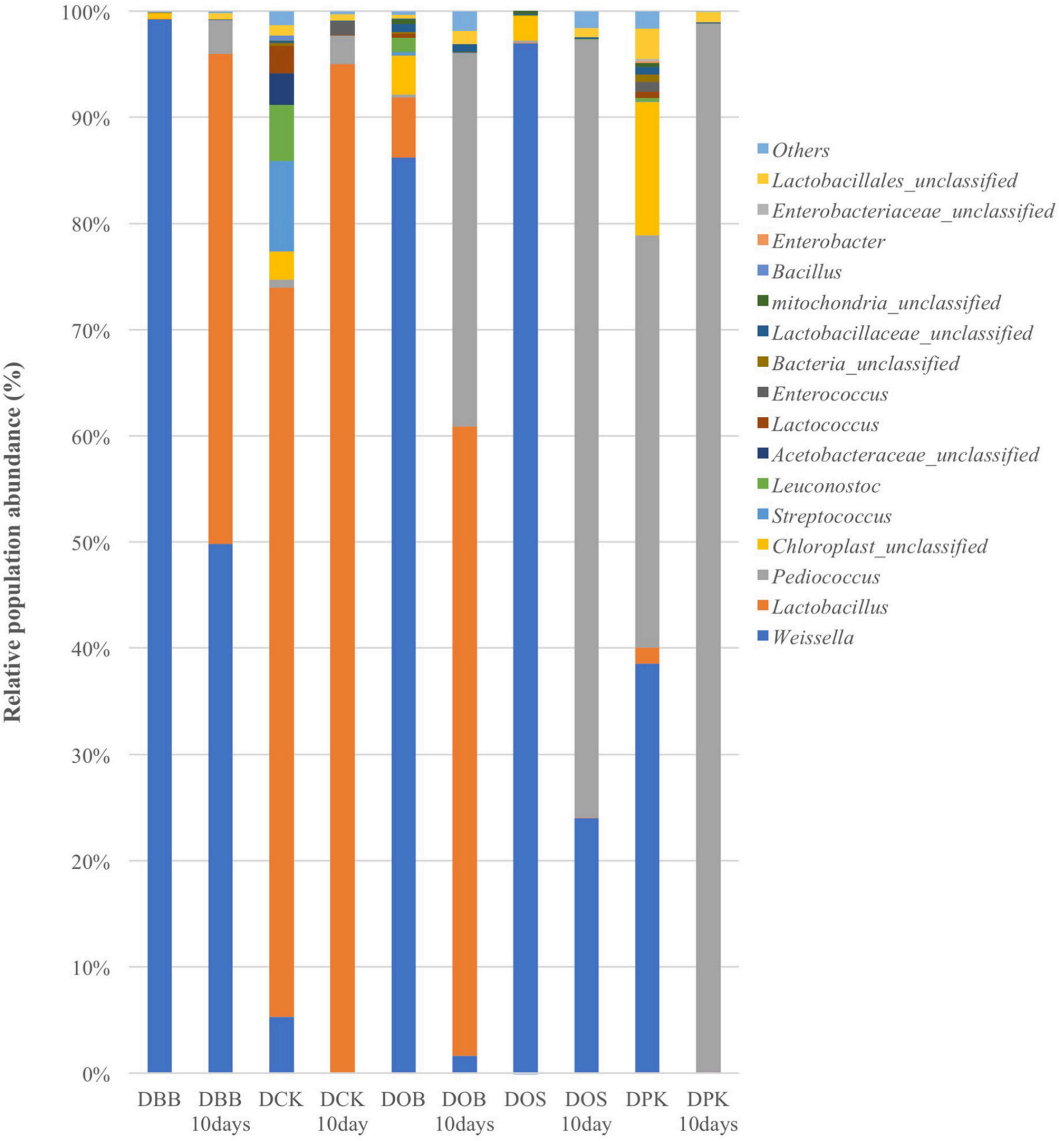
Bacterial composition (OTUs at the genus level based on 16S amplicon sequencing) of the five starters and the corresponding microbial communities after 10 days of fermentation (labeled “10 Days”). DBB, DCK, DOB, DOS, and DPK are the five traditional Cambodian starters.

**Figure 2:**
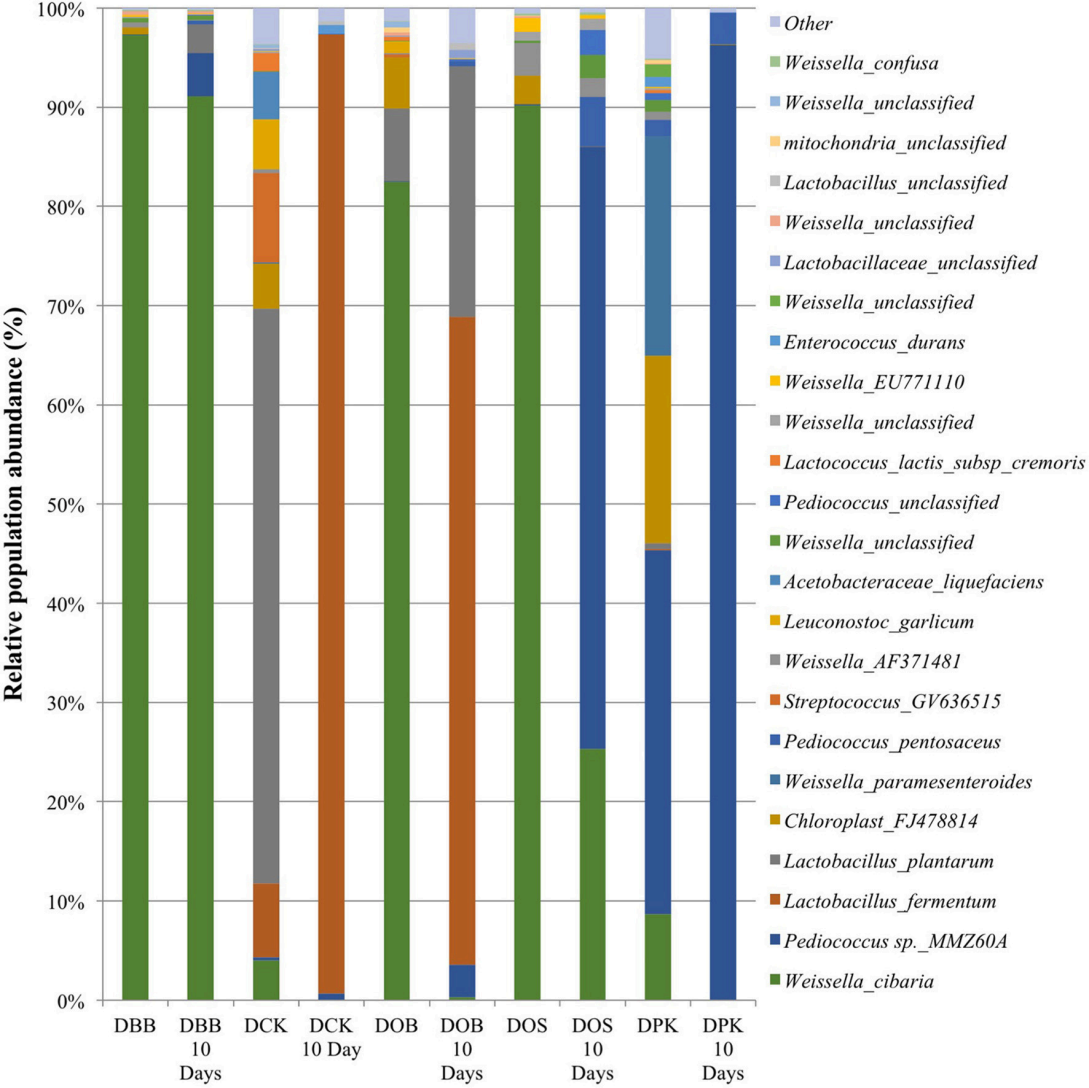
Bacterial composition (OTUs at the species level based on 16S amplicon sequencing) of the five starters and the corresponding microbial communities after 10 days of fermentation (labeled “10 Days”). DBB, DCK, DOB, DOS, and DPK are the five traditional Cambodian starters.

The Cambodian traditional rice wine brewing process has been adapted at a lab scale. Five different Cambodian traditional starters were analyzed as well as the microbial communities resulting from 10 days of fermentation. In this study, the genus and species labeling was addressed based on the V1–V3 region. The relative abundance of each genus and species was compared. As shown in Table 1, in terms of the overall species richness, the DPK dried starter showed the highest species abundance followed by DCK, DOB, DOS, and DBB. Species richness represents the number of different species found in ecological community. The bacterial richness of DPK and DOB dropped from 166.33 and 156.41 in the dried starter to 18.09 and 90.28; respectively, after the fermentation of 10 days. However, the bacterial richness of DOB, DOS, and DBB increased slightly from 93.58, 49.17, and 23.73 to 95.51, 56.67, and 27.51; respectively. This showed that there were considerable changes in terms of bacterial species in the community after the fermentation stage for all type of starters. The microbiota composition of each dried starter (before and after the fermentation) is presented at a genus level (Figure 1) and a species level (Figure 2). According to biplot principal component analysis, the duplicate samples remain close each other while each sample series is far from each other (Figure S1). This demonstrated that microbial composition of the dried starter was specific to the starter considered. The pyrosequencing analysis revealed that most bacterial genera were lactic acid bacteria including *Weissella* (ranging from 35 to 99% of the OTUs), *Lactobacillus* (ranging from 0 to 66% of the OTUs)*, Pediococcus* (ranging from 0 to 39% of the OTUs), *Streptococcus* (ranging from 0 to 9% of the OTUs) and *Leuconostoc* (ranging from 0 to 5% of the OTUs). Large changes in bacterial community have been observed between the dried starters and the microbial communities after fermentation (Figure 2). During fermentation with the DBB starter, *Weissella cibaria*, which was prevalent in the starter, decreased slightly from 96.29 to 91.09% of the OTUs. However, *Pediococcus sp. MMZ60A* and *Lb. plantarum* considerably increased after the brewing process. Similarly, *Lb. plantarum* was found to be dominant in the DCK starter (57.93% of the OTUs) but not detected after the fermentation. Nevertheless, *Lb. fermentum* became prevalent (96.70% of the OTUs). In this starter, several species *Streptococcus GV636515* (9%), *Leuconostoc garlicum* (5%), and *Acetobacteraceae liquefaciens* (4.7%) disappeared after the fermentation. In the DOB consortium, *W. cibaria* was prevalent. After the fermentation, *Lb. fermentum* and *Lb. plantarum* were dominant with respective OTU percentages of 65.29 and 25.24%. *Pediococcus sp. MMZ60A* was prevalent in rice wine after the fermentation stage performed by the consortia DOS and DPK. There was a remarkably impact on the bacterial community in DPK. *Pediococcus sp. MMZ60A* was present at 36.72% and got dominant (96.27% of the OTUs) after the fermentation. Moreover, *Lb. plantarum* was less detected in the dried starter DCK. However, it became the dominant bacterial species after the traditional fermentation (96.70%) while *Lb. fermentum* was detected (57.93% of the OTUs) in the dried starter and not detected after the fermentation. All these changes showed that the distribution of bacteria varied and changed after the fermentation according to the traditional Cambodian process.

**Table 1:** Bacterial diversity, bacterial richness and bacterial evenness of the five starters and the microbial communities after 10 days of fermentation.

**Group**	**Bacterial diversity**	**Bacterial Richness**	**Bacterial Evenness**
DBB	1.04	23.73	0.06
DBB 10 Days	1.18	27.51	0.06
DCK	2.67	156.41	0.03
DCK 10 Days	1.05	90.28	0.03
DOB	1.41	93.58	0.03
DOB 10 Days	1.98	95.51	0.03
DOS	1.20	49.17	0.04
DOS 10 Days	2.22	56.67	0.09
DPK	4.25	166.33	0.05
DPK 10 Days	1.06	18.09	0.09

### Fungal community presented in cambodian traditional dried starter

**Figure 3:**
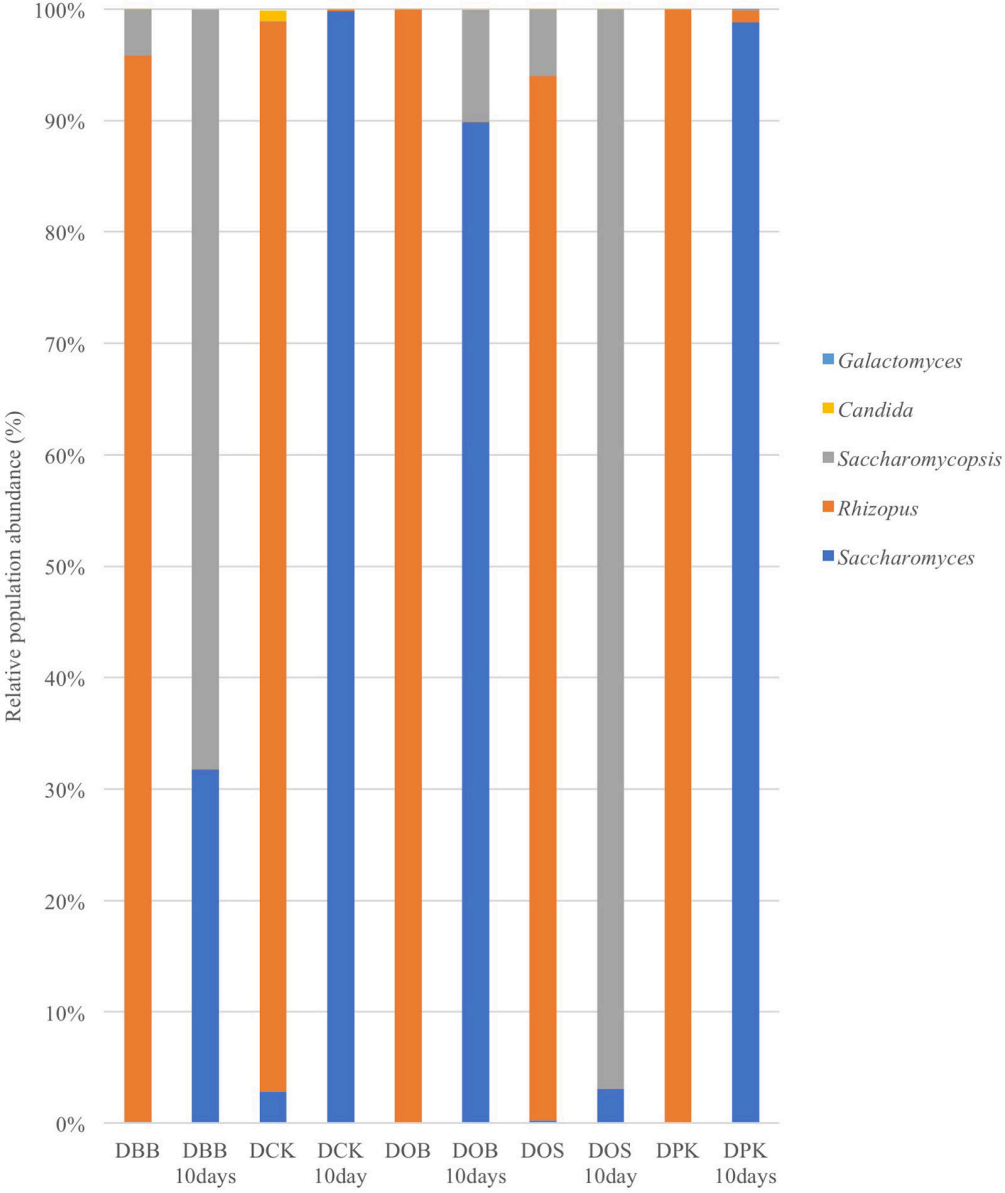
Fungal composition (OTUs at the genus level based on 26S amplicon sequencing) of the five starters and the corresponding microbial communities after 10 days of fermentation (labeled “10 Days”). DBB, DCK, DOB, DOS, and DPK are the five traditional Cambodian starters.

**Figure 4:**
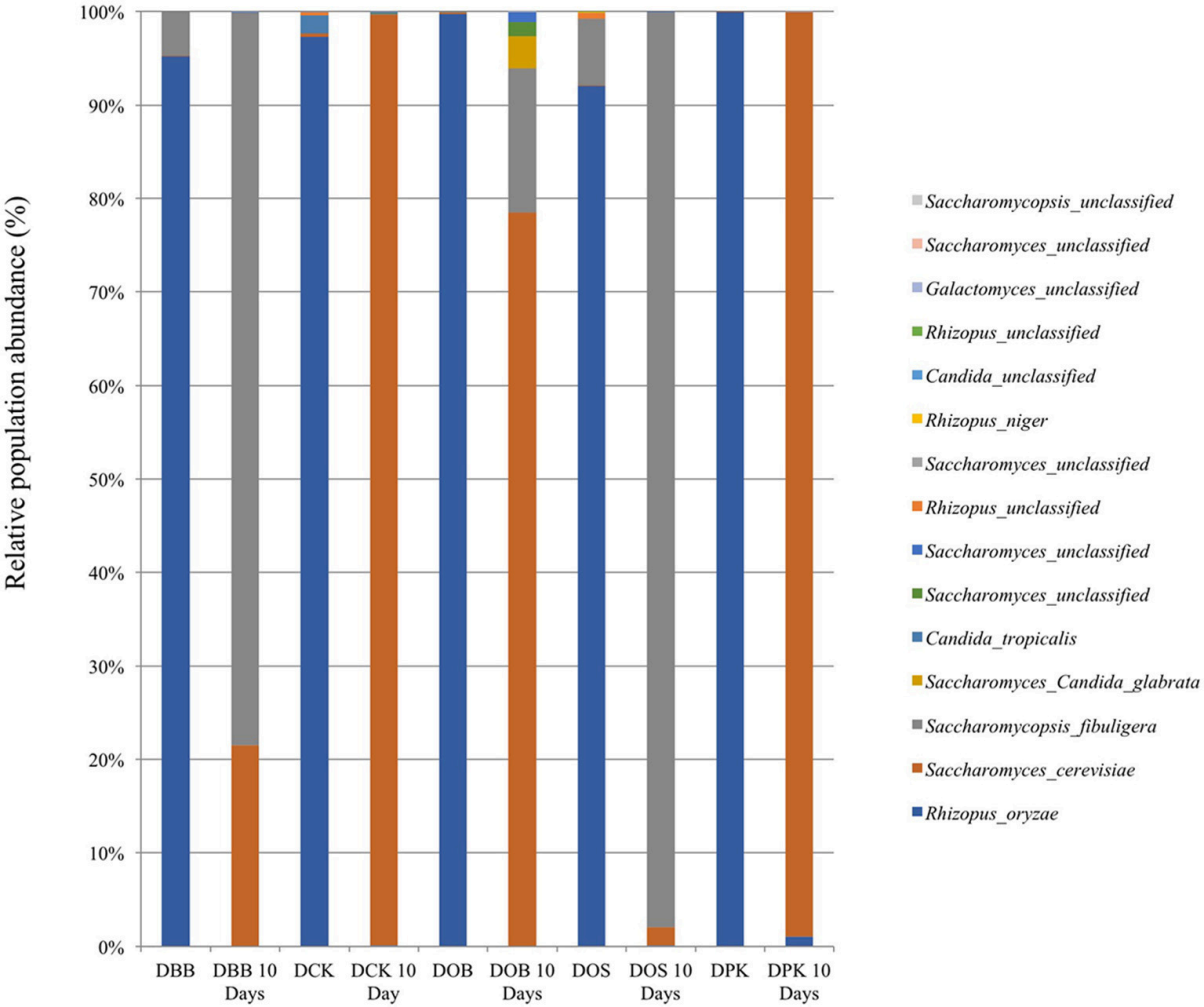
Fungal composition (OTUs at the species level based on 26S amplicon sequencing) of the five starters and the corresponding microbial communities after 10 days of fermentation (labeled “10 Days”). DBB, DCK, DOB, DOS, and DPK are the five traditional Cambodian starters.

The fungal composition (in terms of OTUs) of the starters (before and after 10 days of brewing) is presented at the genus level (Figure 3) and the species level (Figure 4). Once more, strong modifications were observed. According to the results presented in Table 2, there were not much differences of fungal richness in the different types of dried starters. Therefore, it is believed that there were not many fungal species associated in the starter communities. After the fermentation stage, the rice wine obtained with the DCK and DOB starters led to the highest fungal richness (9.86 and 7.13, respectively). The fungal evenness, which refers to the uniformity of the species inside the microbial community, was quite stable in each dried starter and also after the fermentation process. As shown in (Figure 3), the *Rhizopus* genus was found ubiquitously as predominant (ranging from 93 to 99% of the OTUs) in the dried starter; however, it decreased intensely after fermentation. *Saccharomyces* and *Saccharomycopsis* genus became dominant after fermentation depending to their higher presence in raw ferment starter. More species were observed in the communities after 10 days of fermentation comparing to corresponding traditional dried starters (Figure 4 and Table 2). *Rhizopus spp*. was the only filamentous and amylolytic fungal genus found in all dried starters. *Rhizopus oryzae* was the predominant and represented more than 90% of OTUs in each dried starter.

**Table 2:** Fungal diversity, richness and evenness values in the five starters and in the fungal communities after 10 days of fermentation.

**Group**	**Fungal Diversity**	**Fungal Richness**	**Fungal Evenness**
DBB	1.081	2.682	0.425
DBB 10 Days	1.469	3.362	0.488
DCK	1.042	4.836	0.220
DCK 10 Days	1.003	9.866	0.152
DOB	1.001	3.000	0.335
DOB 10 Days	1.506	7.130	0.245
DOS	1.147	5.863	0.222
DOS 10 Days	1.030	4.279	0.278
DPK	1.000	1.228	0.886
DPK 10 Days	1.014	3.441	0.339

### Carbohydrate consumption and ethanol production during the traditional fermentation with five various starters

**Figure 5:**
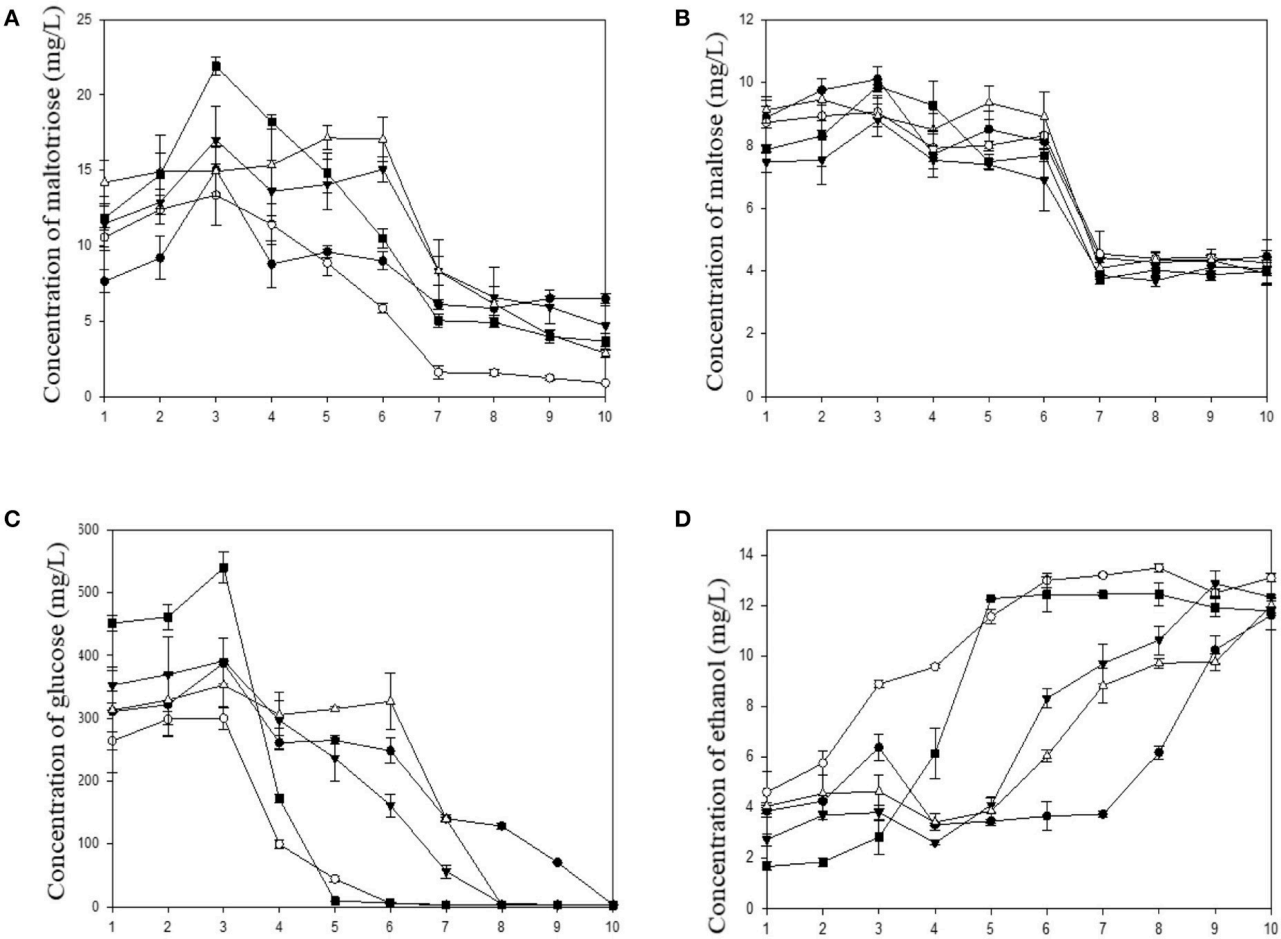
Kinetic of carbohydrate consumption and ethanol production during fermentation based on five microbial starters. **(A)** Maltotriose, **(B)** Maltose, **(C)** Glucose, and **(D)** Ethanol concentration. (DBB, ^•^DCK, °DOB, ^▴^ DOS, and ^■^ DPK).

In this study, sugars and ethanol were measured every 24 h. The profiles of sugar consumption and ethanol production are shown in Figure 5. In rice wine production, the immersion of rice in water and the steam cooking steps are believed to play a role in the breaking down of the structure, to accelerate starch gelatinization and to sterilize rice from microbial agents. According to the results of the microbial community above, *Rhizopus* spp. was associated in the five starters. The presence of this species illustrated that amylolytic enzymes were produced during the brewing process. *R. oryzae* was reported as a strong amylase producer frequently found in amylolytic fermentation starters for rice wine (Dung et al., 2007; Xie et al., 2007; O'Brien and Wang, 2008; Thanh et al., 2008), and was found frequently during traditional fermentation process of Hong Qu glutinous rice wine (Lv et al., 2015). Amylolytic enzymes hydrolyze starch in smaller molecules. In this work, maltotriose and maltose were detected but in small quantities. The profiles of maltotriose and maltose are shown in Figures 5A,B, respectively. The concentrations in these two products reached maximal values at the third day due to the solid state fermentation (steamed red rice with a moisture content approximately of 62%). Some liquid production was observed during this solid state fermentation. Water was added to induce the alcoholic fermentation. At the end of fermentation, maltotriose and maltose were still present and gave rice wine a sweet taste. Interestingly, glucose was much more produced during this brewing process (Figure 5C). The highest concentration in glucose reached a maximal value (from 300 to 550 mg/L) at the third day in all fermentation cases. After 8 days of fermentation, there was no more glucose except in the sample of the DBB starter which ended the fermentation at the tenth day. The results highlighted that there has been a production and a consumption of sugar simultaneously during this brewing. This was due to the presence of amylolytic filamentous fungi and yeasts present in all ferment starters. The evolution of glucose consumption was correlated with the ethanol production. Since the first day of brewing, ethanol was produced in slight concentration (ranging from 2 to 5% v/v). At the fourth day, the concentration slightly decreased because water was added to boost the alcoholic fermentation. It has been observed that the brewing with the DCK and DPK starters occurred faster. Glucose was totally consumed after 6 days and the ethanol production was maximal at the same time. This was due to the predominance of *Saccharomyces cerevisiae* in these starters. However, the final ethanol concentrations were almost similar (between 11.6 and 13% v/v). The final concentration in ethanol at the end of fermentation in this study was similar to the study of Liu et al. (2014).

### Volatile compounds produced by the starters

Twenty-five aromatic compounds were identified by matching to MS library spectra and matching calculated retention time index (RI) values to literature values. The fermentation of red rice wine was made in three replicates in the same conditions with the five starters. The analysis of aromatic compounds was performed in biological triplicate using SPME-GCMS. SPME has been widely used as a method to determine volatile aromatic compounds in rice wine (Ha et al., 2014; Jung et al., 2014; Xiao et al., 2014). A previous study reported that DVB/CAR/PDMS fiber was applicable to the detection of a wide range of aromas in beer, which is also a cereal based beverage (Rodrigues et al., 2008). As results, Table 3 showed the twenty-five compounds identified including esters, alcohols, acids, aldehydes and ketones. Amongst the quantified volatile compounds, the most abundant group was alcohols (about 93% of the total aromatic compounds). As shown in Table 3, 2-methylbutan-1-ol, 3-methylbutan-1-ol, butane-2,3-diol and 2-phenylethan-1-ol were the main volatile compounds. The 3-methylbutan-1-ol was found to be the dominant volatile compound in the different samples (around 54% w/v). The 2-methylpropanol, with a pleasant whiskey flavor, was detected in higher concentrations in the DBB rice wine sample (5976.46 μg/L) and in the DOB sample (5076.98 μg/L) while the concentration was lower in the DOS and DCK samples. Another floral aromatic compound, 2-phenylethan-1-ol, was also found as the third major compound in the five rice wines. Rice wine fermented with DPK showed the highest 2-phenylethan-1-ol production amongst those rice wines with a concentration of 3,624.76 μg/L while the lowest concentration was found in the DCK sample with only 1,607.37 μg/L. Butan-2,3-diol was described as a fruity aroma and was also identified in each rice wine. There were numerous by-products stemming from alcohol fermentation including this compound. It was considered the second most abundant potential source of aroma. The only aldehyde identified and quantified was acetaldehyde. The 2-phenylethylacetate was only found in the rice wine fermented by starter DBB. It is a colorless liquid with a rose flavor that contributes to “rose,” “honey,” “fruity,” and “flowery” aroma nuances (Swiegers et al., 2005). Only three ketones were identified in this study including octan-2-one, 3-hydroxybutan-2-one, and acetophenone.

**Table 3:** Volatile compounds identified in the Cambodian traditional red rice wine after 10 days of fermentation.

**Compounds**	**RI Cal**	**RI Lit^a^**	**DBB**		**DCK**	**DOB**		**DOS**		**DPK**		
			**Means**	**SD**	**Means**	**SD**	**Means**	**SD**	**Means**	**SD**	**Means**	**SD**
**ESTERS**												
Ethyl lactate	1,360	1,358	UD		292.43	6.64	483.90	57.55	241.00	13.39	353.35	7.51
Ethyl acetate	901	898	12.41	0.91	17.50	0.09	28.14	2.78	17.62	0.90	UD	
2-Phenylethyl acetate	1,828	1,837	58.69	11.14	UD		UD		UD		UD	
Ethyl oleate	2,492	2,489	UD		UD		27.37	1.82	10.79	0.95	68.65	4.33
∑			71.11		309.93		539.40		269.41		422.00	
**ALCOHOLS**												
Propan-1-ol	1,049	1,037	1,176.71	135.52	815.01	56.50	1,762.73	15.93	731.38	49.23	423.41	44.40
2-Methylpropan-1-ol	1,072	1,099	5,976.46	378.32	3,668.25	557.68	5,076.98	850.15	3,507.64	308.37	4,399.94	111.80
Butan-1-ol	1,184	1,145	56.26	5.40	34.50	2.34	63.50	2.85	49.75	5.39	63.81	3.73
3-Methylbutan-1-ol	1,237	1,205	13,781.86	1, 588.87	10,077.08	1, 385.76	17,546.66	1, 472.95	10,235.16	694.25	16,320.50	2, 265.41
Pentan-1-ol	1,269	1,255	17.11	1.10	16.49	1.58	17.78	1.97	16.73	1.16	26.73	1.78
Hexan-1-ol	1,362	1,360	87.76	8.32	UD		75.48	3.47	54.85	5.38	UD	
3-Ethoxypropan-1-ol	1,374	1,376	20.43	3.03	28.25	0.01	22.39	1.70	21.46	2.76	22.96	1.04
Heptan-1-ol	1,460	1,467	14.93	1.40	15.93	1.29	18.04	0.27	10.72	0.11	18.97	0.02
Butane-2,3-diol	1,548	1,523	1,067.14	104.64	1,483.15	161.96	1,716.60	127.39	1,146.23	61.66	2,230.13	168.35
Octan-1-ol	1,559	1,553	UD		UD		34.63	2.80	36.97	4.23	16.87	1.03
2-methoxyphenol	1,877	1,875	40.45	5.45	95.01	0.97	56.66	2.43	23.14	0.99	38.69	2.11
Phenylethanol	1,891	1,865	8.80	0.77	17.30	1.01	23.38	1.34	8.24	0.88	15.71	1.66
2-Phenylethan-1-ol	1,928	1,925	2,302.53	315.41	1,607.37	29.65	2,228.61	27.35	2,318.04	29.65	3,624.76	140.48
∑			24,550.45		17,858.35		28,643.43		18,160.30		27,202.48	
**ACIDS**												
Acetic acid	1,470	1,450	633.46	31.22	1,854.84	8.25	766.58	55.86	638.96	82.99	546.93	35.79
2-methylpropanoic acid	1,584	1,563	UD		35.35	2.82	UD		83.66	0.06	91.38	8.28
Octanoic acid	2,088	2,083	18.07	1.75	48.59	5.49	188.59	9.32	10.48	0.38	23.23	2.96
Butanedioic acid	1,680	1,619	63.72	2.64	67.14	1.24	87.83	5.34	20.88	0.66	13.95	0.29
∑			715.25		2,005.92		1,043.00		753.98		675.49	
**ALDEHYDES AND KETONES**												
Acetaldehyde	691	690	116.91	11.99	72.84	4.93	73.66	1.08	207.14	3.45	339.28	44.12
Octan-2-one	1,295	1,285	40.45	4.51	24.08	2.24	32.00	2.72	35.94	1.32	32.70	0.29
3-Hydroxybutan-1-one	1,310	1,295	84.20	5.46	33.14	1.60	34.72	0.81	106.32	7.04	56.10	0.69
Acetophenone	1,664	1,645	18.98	4.03	13.33	0.53	UD		UD		UD	
∑			260.54		143.40		140.38		349.40		428.07	
Total aroma profile			25,597.35		20,317.59		30,366.22		19,533.09		28,728.04	

*UD, Under the detection threshold*,

a *Litterer source http://www.pherobase.com/ Values are expressed as μg/L and are the average of 3 biological repeats ± standard deviation*.

### Correlation between volatile compound and bacteria and fungi species

**Figure 6:**
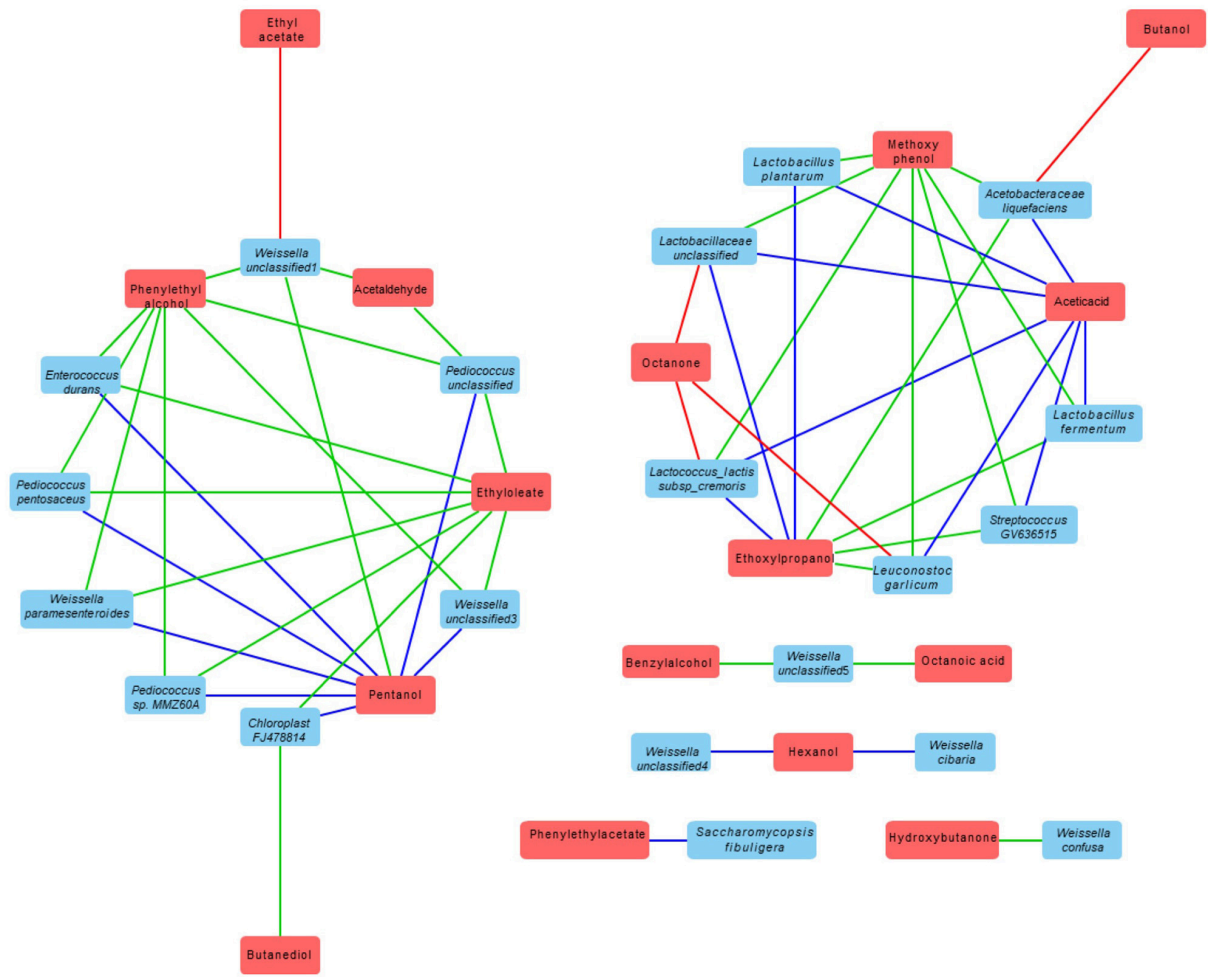
Correlation network between volatile metabolites and microbial starters (based on Cytoscape software). The red boxes represent volatile metabolites and blue boxes correspond to microbial strains that are correlated with this metabolite. The blue line represents the positive correlation with a level of significance of 0.01, the green line represents the positive correlation with a level of significance of 0.05 and the red line represents the negative correlation with a level of significance of 0.05.

The correlation between the volatile compound and bacteria and fungi species presented in each dried starter is shown in Table 4. Cytoscape Network software was used for visualizing the interaction and correlation (Figure 6). Only the correlation coefficient significant at least at 0.05 level were discussed in this part. The correlation coefficient indicated a very strong relation (from 0.882 to 1). The complexity of variety of microbial community have generated intricate and specific aromatic profiles. Relatively high and significant correlations with volatile compound produced were observed with the presence of various strains including mostly *Weissella* genus; *Weisella cibaria, Weissella paramesenteroides, Weisella confusa, Weisella unclassified, Acetobacteraceae liquefaciens, Lactobacillus plantarum, Lactobacillus fermentum, Lactobacillaceae unclassified, Pediococcus sp. MMZ60A, Pediococcus unclassified, Leuconostoc galicum, Lactococcus lactis, Streptococcus GV636515*, and *Saccharomycopsis fibuligera*. Phenyl ethylalcohol, a pleasant floral odor; benzyl alcohol, mild pleasant aromatic odor, were strongly correlate with the most of *Weissella* and *Pediococcus* genus. Phenyl ethylacetate was found to be perfect correlated with only *Saccharomycopsis fibuligera*. Negative relation of octanone were observed with the presence of *Lactococcus Lactis, Leuconostoc garlicum, Lactobacillaceae unclassified*, ethyl acetate with *Weisells unclassified1* and butanol with *Acetobacteraceae liquefaciens*.

**Table 4:** Correlation between the volatile compounds produced by each dried starter and bacteria and fungi species presented in each starter.

**Strains**	**Compounds**	**Pearson Correlation coefficient**	***p*-value**
*Saccharomycopsis_fibuligera*	Phenyl ethylacetate	1.000	0.000
*Lactobacillus_plantarum*	Acetic acid	0.997	0.000
*Pediococcus_pentosaceus*	pentanol	0.994	0.001
*Enterococcus_durans*	pentanol	0.994	0.001
*Weissella_unclassified3*	pentanol	0.994	0.001
*Pediococcus sp. MMZ60A*	pentanol	0.993	0.001
*Weissella_paramesenteroides*	pentanol	0.993	0.001
*Streptococcus_GV636515*	Acetic acid	0.993	0.001
*Pediococcus_unclassified*	pentanol	0.993	0.001
*Leuconostoc_garlicum*	Acetic acid	0.992	0.001
*Acetobacteraceae_liquefaciens*	Acetic acid	0.990	0.001
*Lactobacillus_fermentum*	Acetic acid	0.989	0.001
*Lactococcus_lactis_subsp_cremoris*	Ethoxyl propanol	0.988	0.001
*Lactobacillaceae_unclassified*	Ethoxyl propanol	0.987	0.002
*Chloroplast_FJ478814*	pentanol	0.969	0.007
*Lactococcus_lactis_subsp_cremoris*	Acetic acid	0.966	0.007
*Lactobacillaceae_unclassified*	Acetic acid	0.965	0.008
*Weissella_cibaria*	hexanol	0.963	0.008
*Weissella_unclassified4*	hexanol	0.961	0.009
*Leuconostoc_garlicum*	Methoxyphenol	0.960	0.010
*Lactobacillus_plantarum*	Ethoxyl propanol	0.958	0.010
*Weissella_unclassified1*	Phenyl ethylalcohol	0.953	0.012
*Streptococcus_GV636515*	Ethoxyl propanol	0.952	0.013
*Weissella_unclassified5*	Octanoic acid	0.952	0.013
*Lactobacillus_fermentum*	Ethoxyl propanol	0.951	0.013
*Chloroplast_FJ478814*	Ethyl oleate	0.949	0.014
*Acetobacteraceae_liquefaciens*	Ethoxyl propanol	0.949	0.014
*Leuconostoc_garlicum*	Ethoxyl propanol	0.948	0.014
*Lactococcus_lactis_subsp_cremoris*	methoxyphenol	0.948	0.014
*Lactobacillus_plantarum*	methoxyphenol	0.939	0.018
*Weissella_unclassified1*	Pentanol	0.933	0.021
*Chloroplast_FJ478814*	Butanediol	0.931	0.021
*Lactococcus_lactis_subsp_cremoris*	Octanone	−0.926	0.024
*Lactobacillaceae_unclassified*	Methoxyphenol	0.922	0.026
*Pediococcus_pentosaceus*	Ethyl oleate	0.921	0.026
*Pediococcus_unclassified*	Ethyl oleate	0.921	0.026
*Enterococcus_durans*	Ethyl oleate	0.921	0.026
*Weissella_paramesenteroides*	Ethyl oleate	0.920	0.027
*Pediococcus sp. MMZ60A*	Ethyl oleate	0.919	0.027
*Pediococcus_unclassified*	Phenylethyl alcohol	0.919	0.027
*Weissella_unclassified3*	Ethyl oleate	0.919	0.027
*Weissella_confusa*	Hydroxy butanone	0.919	0.027
*Weissella_unclassified3*	Phenylethyl alcohol	0.918	0.028
*Pediococcus_pentosaceus*	Phenylethyl alcohol	0.917	0.029
*Enterococcus_durans*	Phenylethyl alcohol	0.917	0.029
*Weissella_paramesenteroides*	Phenylethyl alcohol	0.915	0.029
*Pediococcus_EU157914*	Phenylethyl alcohol	0.914	0.030
*Streptococcus_GV636515*	methoxyphenol	0.913	0.030
*Weissella_unclassified1*	Ethyl acetate	−0.911	0.031
*Weissella_unclassified1*	Acetaldehyde	0.908	0.033
*Lactobacillus_fermentum*	methoxyphenol	0.903	0.036
*Lactobacillaceae_unclassified*	octanone	−0.902	0.036
*Acetobacteraceae_liquefaciens*	methoxyphenol	0.901	0.037
*Weissella_unclassified5*	benzylalcohol	0.885	0.046
*Leuconostoc_garlicum*	octanone	−0.882	0.048
*Pediococcus_unclassified*	Acetaldehyde	0.882	0.048
*Acetobacteraceae_liquefaciens*	Butanol	−0.878	0.050

## Discussion

This study represents the first attempt using rDNA pyrosequencing to investigate the microbiotas in five different Cambodian traditional dried starters, and to examine the changes of microbial composition after 10 days of fermentation. It has been reported that the microbiota composition of rice wine starter was highly variable (Sujaya et al., 2001; Thanh et al., 2008). The results observed in this study were in agreement with the previous findings of Lv et al. (2013a) and Ramos et al. (2011) at the level of lactic acid bacteria (LAB). The prevalence of LAB in fermented food was commonly due to their ability to tolerate low pH values (Abriouel et al., 2006). This is the reason that potential foodborne pathogens were not detected after having achieved the traditional rice wine fermentation process. The composition of LAB in the starters applied to the production of alcoholic beverages was also investigated by Thanh et al. (2008). Their results showed that *P. pentosaceus, Lb. plantarum, L. brevis, W. confuse*, and *W. paramesenteroides* were detected in Vietnamese starters using a 16S rRNA gene-based PCR-based denaturing gradient gel electrophoresis. However, only the bacterial population that represents at least 1% of the total community would probably be detected by DGGE (Weisburg et al., 1991). Thus, the meta-genomic analysis is a useful tool to investigate the composition of microbial communities since it is capable to detect lower populations. Basically, a spontaneous cereal-based fermentation is induced by the combination of yeasts, fungi and lactic acid bacteria (Blandino et al., 2003). The study of Nout and Sarkar (1999) have shown that the growth of yeasts in fermented food is favored by the acidification caused by bacteria. Another study revealed that *Saccharomyces cerevisiae* adjust its metabolism by secreting a serial metabolite, notably amino acid, allowing the survival of LAB (Ponomarova et al., 2017). The presence of LAB in cereal fermentation is probably crucial because beside producing lactic acid, LAB is likely to contribute production of other flavor compounds (Mukisa et al., 2017). Environmental stress, particularly acid stress; induced the formation of specific aromatic compounds during the lactic acid fermentation (De Angelis et al., 2001; Serrazanetti et al., 2009, 2011). Therefore, the aroma type and its concentration might be determined by the substrate composition, the starter culture and the environmental conditions of the process. The taxonomic analysis has shown a complex bacterial community in the Cambodian dried starters, even after the fermentation stage with red rice as a raw material. Most species were identified as lactic acid bacteria but they varied in different proportions. The genera *Lactobacillus, Leuconostoc, Weissella*, and *Pediococcus* were found on the grains' surface and in the surrounding environment. This is the fact that they are found with fungal strains in fermented cereal based food (Guyot, 2012). LAB are also seen as favorable microorganisms associated with cereal based beverages since it has been shown that they improve protein digestibility, increase nutritional bioavailability and enhance organoleptic quality (Luana et al., 2014). Based on the traditional brewing, the variety of the starters is an important factor influencing both the rice wine flavor and quality. The growth of LAB species during rice wine brewing might affect the growth of yeasts and filamentous fungi, which also contributes to the flavor of rice wine (Lv et al., 2013a). To notice that the locally produced dried starters by rice wine producers could be different based on their individual methods and specific ingredients from one to another region. This variation might therefore affect the starters' quality in terms of final composition of the microbial consortia found in the starters.

There were changes in fungal diversity after 10 days of fermentation, at both levels of filamentous fungi and yeast species. This might be due to the predominance of species in starter, the decreasing pH induced by the LAB and the protocol of starter preparation. The microbial composition of starters varied according to the regions considered, the environment and the material used. According to the study of Yamamoto and Matsumoto (2011), traditional dried starters have widely been used for rice fermentation in Cambodia. Herbs and spices were used as ingredients for the production of dried starters including ginger, chili, pepper, cloves etc. Mixing cultures with spices and oriental herbs were believed to prevent the growth of unfavorable microorganisms and to enhance the synthesis of interesting aromas. Many studies reported various fungi and bacterial species in starters (Aidoo et al., 2006; Dung et al., 2006; Jeyaram et al., 2008; Thanh et al., 2008). The study of Dung et al. (2005) focused on the effect of each oriental ingredient frequently added to dried starters in Vietnam. This study revealed that various herbs and spices have a great impact on biomass and the yeasts during the fermentation. In Cambodia, both dried starters and rice wine preparations are done in an open environment. This leads to increase the microbial diversity. This process must also ensure a good organoleptic quality of the final product. The flavor profile is the most important characteristic of rice wine and can be affected by the consortium of microorganisms used. It has been shown that the flavor of rice wine could be changed and increased when the fermentation process is performed by non*-Saccharomyces* species (Medina et al., 2013). The behavior of *R. oryzae* was observed and its ability to produce volatile compounds during fermentation such as ethanol, 2-methylpropanol and 3-methylbutanol was highlighted (Bramorski et al., 1998; Christen et al., 2000). These two last compounds were the major aromatic molecules produced by the five starters (Table 3). Each dried starter contained *Saccharomyces cerevisiae* and *Saccharomycopsis fibuligera*. However, the yeast specie which was prevalent in the dried starters became dominant after 10 days of fermentation. For example, in the cases of the dried starters DBB and DOS, *S. fibuligera* got dominant (final proportion of 78.39 and 97.92% of OTUs, respectively) while this species was found in high proportions in the original ferment starters. In the starters DCK, DOB, and DPK, *S. cerevisiae* was the only fermenting species in OTUs' proportions of 99, 78, and 98%, respectively. The DCK and DPK starters containing only *S. cerevisiae* as the prevalent species performed the fermentation faster than the other dried starters which contained *S. fibuligera* alone or in combination with another fermenting species. This performance was observed due to the glucose consumption and ethanol production speed (Figure 5). However, the final concentrations in ethanol were not significantly different after 10 days of fermentation (between 11.6 and 13% v/v). The presence of *S. cerevisiae* and *S. fibuligera* was in good agreement with the study of Lv et al. (2013b) which studied on yeast diversity in Chinese traditional starters. This study provided evidence that each microorganism plays a role in the consortium, and therefore affects the final quality of the product derived from the fermentation process. A similar study of (Sha et al., 2017) revealed *Marcha* and *Thiat*, ferment starters in India and Nepal, are composed of different fungal communities*. S. cerevisiae* produces small quantities of 3-methylbutan-1-ol under fermentative condition at low pH. *S. cerevisiae* generate L-leucine via pyruvate metabolism, and 3-methylbutan-1-ol is generated via the L-leucine degradation III pathway. This compound provides wine with a malt-like odor. In Chinese rice wine (Xiao et al., 2014), guava wine (Pino and Queris, 2011) and cherry wine (Dung et al., 2005; Niu et al., 2011), esters were found to be the major volatile compounds. Acetate esters and ethyl esters of fatty acids are formed by the reaction of an organic acid with alcohol during the fermentation, leading to fruity aromas in wine (Villamor and Ross, 2013). However, in this study, the alcohol group was predominant. It could be due to the absence of reactions between carboxylic acids and alcohols. Another reason is because of freshly harvesting and analyzing SPME-GCMS quite immediately after fermentation to see the different flavor compound produced by the communities. In general, most flavor compounds, especially esters in rice wine, are principally produced after fermentation (Wang et al., 2014). The aromas' types and their concentrations might be influenced once more by the substrate composition, the starter culture, the environmental conditions and the process applied. Some species presented in small quantity in the community still have strong correlation with volatile compounds. It was found that *Weissella, Pediococcus*, and *Lactobacillus* genus has most mutually related with flavor compounds. During the fermentation process with starter DCK, the *Lb. plantarum* species decreased while it increased in the fermentation with starter DOB. However, DCK and DOB starter exhibited a different initial microbial composition. One possible explanation is that DCK starter contained the yeast *S. cerevisiae* as a predominant species. Accordingly, alcoholic fermentation was more intensive when using this starter, leading to inhibiting conditions for the other species. The bacterial community of DOB starter, *Lb. fermentum* and *Lb. plantarum* were found as dominant at the end of fermentation while the volatile compound was hugely produced. *Lactobacillus* is an important genus involved in grape fermentation. *Lb. plantarum* is found frequently on grape and in wine and is often involved in spontaneous malolactic fermentation. Recently, some researchers have revealed that *Lb. plantarum* species shows a different enzymatic profile from other LAB species, which could play an important role in the wine aroma profile (Swiegers et al., 2005; Lerm et al., 2011; Iorizzo et al., 2016). The interaction between LAB and yeasts has been known to enhance the growth of either group of microbes (Mugula et al., 2003; Omemu et al., 2007) and to build up the alternative flavor production (Mukisa et al., 2017). This study highlighted the variable pattern structure of microbiota in the spontaneous red rice wine fermentation. The variable categories and concentrations of the flavor compounds were intensely affected by the nature of these microbial communities. Competitive metabolic interactions among species often play a critical role in the structure and the functions of multispecies communities. However, metabolic interactions still play an important role in regulating microbial activities and in maintaining the diversity in microbial communities during the brewing process itself. The results presented here fully enrich our understanding of the microbial community exploited in rice wine brewing and the corresponding aromatic profiles. Further studies should be performed to understand the interactions between LAB, yeasts and molds to define the most important factors contributing to the final flavor of rice wine.

## Author contributions

SL performed the main experiments and drafted the manuscript. HM performed duplicates experiments and reviewed the manuscript. CT interpreted amplicon sequencing data. BT and GD performed amplicon sequencing analyses. M-LF performed SPME-GC-MS data analysis. FD designed the experiments and drafted the manuscript.

### Conflict of interest statement

The authors declare that the research was conducted in the absence of any commercial or financial relationships that could be construed as a potential conflict of interest.

## Supplementary material

Figure S1 Biplot of both bacterial and fungal composition of five ferment starters. According to the biplot principal component analysis, the duplicate samples stay near each other this shows that the samples were quite replicable. Moreover, each sample series stays far from each other.
