# Supplementary figures and images for: Impact of Microbial Composition of Cambodian Traditional Dried Starters (Dombea) on Flavor Compounds of Rice Wine: Combining Amplicon Sequencing With HP-SPME-GCMS
Source: Front Microbiol. 2018 May 8;9:894. doi: 10.3389/fmicb.2018.00894 (PMC5951977; doi:10.3389/fmicb.2018.00894)

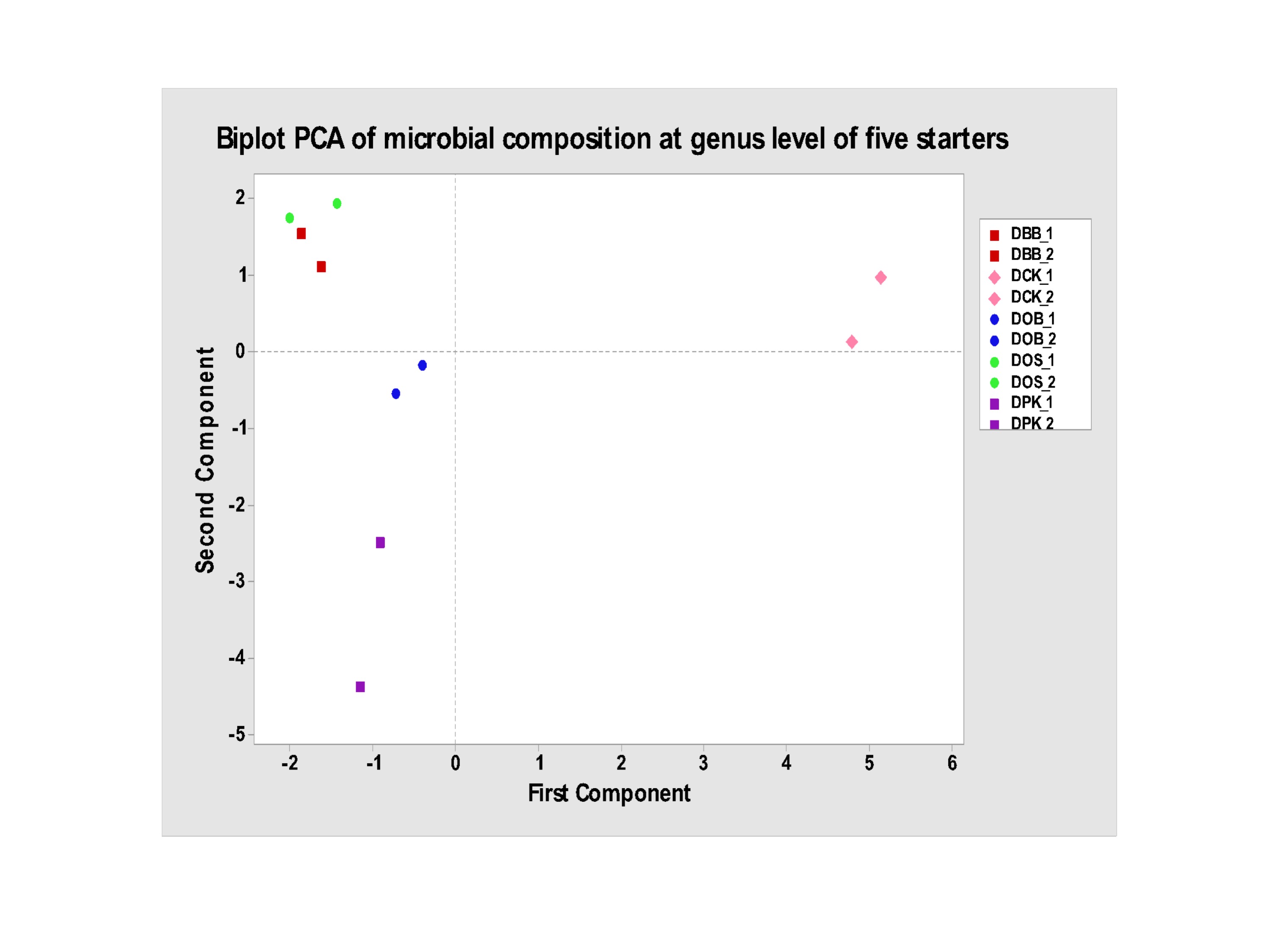

Supplement: Figure S1 — Biplot of both bacterial and fungal composition of five ferment starters. According to the biplot principal component analysis, the duplicate samples stay near each other this shows that the samples were quite replicable. Moreover, each sample series stays far from each other. [file Image_1.jpg]
